# Cold Plasma—A Sustainable Energy-Efficient Low-Carbon Food Processing Technology: Physicochemical Characteristics, Microbial Inactivation, and Industrial Applications

**DOI:** 10.1155/ijfo/4166141

**Published:** 2025-03-16

**Authors:** Ramesh Sharma, Pinku Chandra Nath, Sarvesh Rustagi, Minaxi Sharma, Baskaran Stephen Inbaraj, Praveen Kumar Dikkala, Prakash Kumar Nayak, Kandi Sridhar

**Affiliations:** ^1^Department of Food Technology, Sri Shakthi Institute of Engineering and Technology, Coimbatore, India; ^2^Research and Development Cell, Manav Rachna International Institute of Research and Studies (Deemed to Be University), Faridabad, Haryana, India; ^3^Department of Food Technology, Uttaranchal University, Dehradun, Uttarakhand, India; ^4^Research Centre for Life Science and Healthcare, Nottingham Ningbo China Beacons of Excellence Research and Innovation Institute (CBI), University of Nottingham Ningbo China, Ningbo, China; ^5^Department of Food Science, Fu Jen Catholic University, New Taipei City, Taiwan; ^6^Department of Food Technology, Koneru Lakshmaiah Education Foundation, Vaddeswaram, Guntur, Andhra Pradesh, India; ^7^Department of Food Engineering and Technology, Central Institute of Technology Kokrajhar, Kokrajhar, India; ^8^Department of Food Technology, Karpagam Academy of Higher Education (Deemed to Be University), Coimbatore, India

**Keywords:** cold plasma, microbial inactivation, physicochemical properties, preservation, reactive species

## Abstract

Nonthermal technologies, mostly utilized for microbial inactivation and quality preservation in food, are attracting increased interest, particularly in nonthermal plasma. Cold plasma (CP) demonstrates favorable results, such as increased germination, enhanced functional and rheological characteristics, and the eradication of microorganisms. Consequently, CP is a novel technology in food processing that has significantly contributed to the prevention of food spoilage. This study highlights contemporary research on CP technology in food processing. This includes its use in microbial decontamination, shelf life extension, mycotoxin degradation, enzyme inactivation, and surface modification of food products. The CP generation techniques under low pressure, including glow discharge, radio frequency and microwave techniques, and atmospheric pressure, including dielectric barrier discharge (DBD), plasma jet, and corona discharge, are discussed. Additionally, the source for the generation of plasma-activated water (PAW) with its significant role in food processing is critically discussed. The CP is an effective method for the decontamination of several food materials like fruits, vegetables, meat, and low-moisture food products. Also, the review addressed the effects of CP on the physicochemical properties of foods and CP for pretreatment in various aspects of food processing, including drying of food, extraction of bioactive compounds, and oil hydrogenation. CP improved the drying kinetics of food, resulting in reduced processing time and improved product quality. Similarly, CP is effective in maintaining food safety and quality, removing the formation of biofilm, and also in reducing protein allergenicity. The review also underscored the importance of CP as a sterilizing agent for food packaging materials, emphasizing its role in enhancing the barrier characteristics of biopolymer-based food packaging materials. Therefore, it is concluded that CP is effective in the reduction of pathogenic microorganisms from food products. Moreover, it is effective in maintaining the nutritional and sensory properties of food products. Overall, it is effective for application in all aspects of food processing. There is a critical need for ongoing research on upscaling for commercial purposes.

## 1. Introduction

New processing techniques that preserve the majority of the quality attributes are the focus of research; these include pulsed electric field, CP, infrared heating, ultrasound, ohmic heating, high-pressure processing (HPP), ozone processing, pulsed light, and dielectric heating (microwave (MW) and radio frequency (RF)). These new methods provide little processing, maintain food's sensory qualities, and shield food's bioactive substances and their ability to function for longer periods. Novel methodologies have multiple applications in the food sector; they can reduce heating and residence times, improve food quality, increase energy yield, modulate Maillard and other chemical processes, and protect against environmental pressures [1].

CP operates by utilizing charged gaseous species and molecules that possess high reactivity to neutralize detrimental microbes present in food products and packing materials [2]. CP is generated from ionized gas with the presence of active particles, including electrons, ions, free radicals (FRs), and UV photons, which are produced by electrical energy sources. The generation of RS can be facilitated by plasma processing factors such as frequency, gas compositions, voltage level, exposure time for plasma, processing time, and geometry of electrodes. Air plasma is considered a potential source for producing reactive oxygen species (ROS) and reactive nitrogen species (RNS). These RS diffuse on material surfaces with physicochemical modification inducing negative or positive changes [3], thereby bringing topography change on the surface of food [4]. This chemical- and water-free technology is thought to be ecofriendly and leaves no residue, which can be used on both solid and liquid food products. The technology is less expensive than conventional technologies since it requires no heat, pressure, or vacuum conditions, and processing durations are quicker in CP [5, 6]. The CP can be used for allergy elimination, pesticide control, microbiological cleansing, and food functioning [7]. This technique showed encouraging results about the safety issues connected to various product types. Studies found that CP-treated plant-based foods had higher antioxidant content. This could be because the CP causes cell membranes to be disrupted by active plasma species, which erode the membrane and release bioactive chemicals. In addition, in model food systems exposed to plasma, enzyme inactivation was accomplished [8]. Results verified that this innovative technique offers promising outcomes in food processing applications on a lab scale.

The pretreatment with CP for drying food showed improved results in terms of drying rate with the addition of a positive effect on food products [9]. CP functions as pretreatment in various food processing sectors, including drying, extraction of phytochemicals, cooking of food, and curing of food products for oil hydrogenation [10]. A cutting-edge method called CP treatment alters the polymer's surface, opening up possibilities for the creation of packaging materials with appropriate properties. Zein and polyacid were pretreated with CP to improve the mechanical, water barrier, and hydrophobic qualities of the films as well as the compatibility and adhesion between the layers [11].

Thus, this review explored the fundamentals of CP, plasma generation under low and atmospheric pressure (AP), plasma-activated water (PAW), and their applications, plasma reactive species (PRS), the effect of CP on the physicochemical characteristics of food, and microbial inactivation of food for its safety and preservation. Also, it highlights the utilization of CP as a pretreatment technique for various fields of food processing, highlighting its applications in the modifications of biopolymers for food packaging.

## 2. Generation of CP

The plasma source types and processing parameters (like pressure, degree of ionization, and gas frequency) are used to determine the reactivity of the plasma [12]. A partially ionized gas known as CP is generated when a neutral gas at atmospheric or low pressure is subjected to sufficient energy sources, including radio frequencies, MWs, thermal energy, electromagnetic fields, and electric fields [13]. The unbound electrons in the gas absorb the given energy more rapidly than the ions. The energy is then conveyed to heavier gas molecules via accelerated electrons via both elastic and inelastic collisions. The gas experiences several phase transformations due to these collisions, including ionization, excitation, and dissociation, resulting in the generation of additional electrons, ions, and reactive species, including reactive nitrogen and oxygen species [14]. CP has comparatively high electron temperatures (1–10 eV), while heavy particles' translational energy is almost at room temperature (molecules and ions) [15]. According to Snoeckx and Bogaerts [16], they found that this is due to electrons possessing a lower mass than more massive particles. As a result, the electrons attain a greater temperature and kinetic energy more quickly compared to heavy particles. Because of this temperature differential between electrons and heavy particles, CP is thus described as being in thermodynamically equilibrium conditions. Compared to thermal plasmas, which require a larger power input (~50 MW), CP requires a far lower power input. Thermal plasmas are produced at high pressures (≥ 105 Pa) and temperatures (4000–≥ 20,000 K). Nonthermal plasma is preferred in the processing of food because of higher energy efficacy and better chemical selectivity. The extent of the chemical process and ionization in nonthermal plasma is controlled by the electron temperature [17].

### 2.1. PRS

The PRS are grouped into long-lived and short-lived reactive species. The long-lived PRS are those that may persist for a considerable amount of time without going through major chemical reactions or breaking down [18]. Certain excited-state molecules, such as singlet oxygen (^1^O_2_), and some FRs, such as nitric oxide (NO), are examples of long-lived PRSs. In contrast, short-lived RSs participate in chemical reactions or transformations quickly, making them extremely reactive and having very short life spans. Because of their extremely erratic or energetic structures, short-lived PRSs frequently react with nearby molecules at a faster rate. High-energy intermediates created during chemical reactions and extremely reactive FRs, such as the hydroxyl radical (•OH), are examples of short-lived PRSs [19].

Helium and argonium are the main gases used in plasma processing to generate the reacting species, which are nonreactive and used for sanitization purposes [20]. ROS include FRs such as superoxide anion (O_2_^●−^), ^●^OH, as well as nonradical molecules like hydrogen peroxide (H_2_O_2_) and ^1^O_2_ [21]. Stepwise reduction of molecular oxygen (O_2_) by high-energy exposure or electron-transfer reactions leads to the production of highly reactive ROS. Similarly, examples of RNS include NO, nitrogen dioxide (NO_2_), nitrite (NO_2_^−^), and nitrate (NO_3_^−^) [22]. Furthermore, the atmospheric air for the generation of ozone is highly recommended in terms of its utilization in the food processing area. In fact, a survey of the quickly growing body of research reveals a distinct tendency favoring the use of air as the preferred CP gas [23]. The FRs generated using air plasma include hydroxyl (HO•), superoxide (O_2_•^−^), alkoxyl (RO•), NO (NO•), and peroxyl (ROO•). The nonradical species generated from air plasma included ozone (O_3_), H_2_O_2_, and ^1^O_2_. Considering various ROS, HO• radicals have higher potential, which is followed by O_3_ and RO• radicals [24].

## 3. CP Types in Food Application

### 3.1. Production of CP at Low Pressure

To generate the CP, either AP or low pressure is used. A low-pressure cold plasma (LP-CP) production component includes a vacuum chamber, a pump (for expelling unnecessary gases through the system), gas supplier tanks, a gas controller, pressure gauges, electrodes, and a MW. A microcontroller (programmable logic controller) can then be used to operate this low-pressure system [25]. Since low-pressure plasma production is operated under vacuum conditions in the enclosed vessel, furthermore, the plasma inside the closed tank is distributed evenly [26]. Low-pressure plasma production can only be used for processing under batch conditions and is not appropriate for continuous systems. The costly vacuum equipment is needed for setting up the instruments [27]. The following describes basic low-pressure plasma productions that are utilized in food processing.

#### 3.1.1. Glow Discharge

Supply of electric current flowing at 100 V through a gas medium over two or more electrodes produces CP in a glow discharge method. This electric current may be of low frequency (50 Hz), DC, AC, or RF with 40 kHz and 13.56 MHz [28]. This type of plasma's electrons obtain enough energy via excitation collisions to produce photons, which are what give this sort of plasma its visual glow [29].

#### 3.1.2. RF

**Figure 1:**
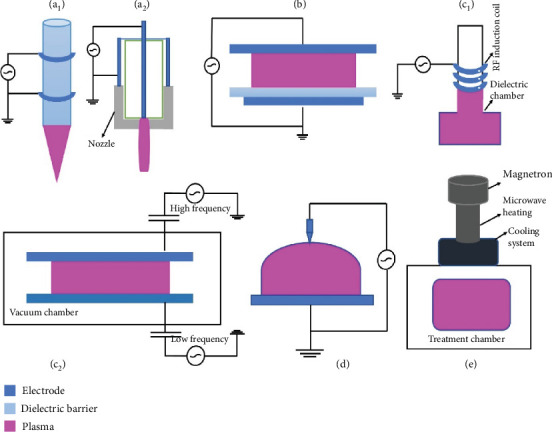
The overall configuration of cold plasma. (a_1_, a_2_) AP plasma jet, (b) AP dielectric barrier discharge (DBD), (c_1_, c_2_) inductively and capacitively coupled plasma, (d) corona discharge, and (e) microwave plasma source.

Inductively coupled plasma (ICP), capacitively coupled plasma (CCP), and helicon wave sources are the three basic forms of RF plasma. The two most popular for industrial applications are ICP and CCP (Figure 1). The CCP setup uses two parallel electrodes in a vacuum chamber that are spaced apart by a few centimetres [30]. With the supply of alternating voltage between two electrodes, RF is created [31]. A roughly 1 kW RF power source that operates at a typical frequency of 13.56 MHz drives the electrodes. In a recent work, 13.56 MHz RF plasma was used to change the structural components of granules in corn starch throughout a relatively brief (10 min) treatment. The helix order of the amylose is improved, and the amorphous areas in the starch granules were destroyed after RF plasma treatment, increasing the maize starch's thermal stability. The adoption of this technology, a unique method for starch modification, could result in the production of high-temperature stable semiconductors and food packaging [32].

#### 3.1.3. MW

A magnetron emits electromagnetic waves, typically at a frequency of 2.45 GHz, which are used to generate MW discharges in MW plasma generators (Figure 1). Without using electrodes, gas molecule electrons are accelerated by the MW electric field with the generation of the CP. At low and ambient pressure, this system is capable of producing plasma. Using an MW frequency of 2.45 GHz and a supply power of 1.1 kW [33] created a pilot-plant scale to create CP-treated water. The water that resulted was used to wash freshly cut lettuce, which caused the bacteria count to drop by about 2 log. The fact that 1.500 L of water from the pilot plant's batch mode was used to disinfect 45 kg of freshly cut lettuce shows the technology's significant potential for usage in the production of vegetables.

### 3.2. Production of Cold Plasma at AP

Corona discharge (CD) plasma systems, dielectric barrier discharge (DBD) plasma systems, and AP plasma jets (PJs) are examples of plasma types that function under AP conditions. It could have either DC or AC power. Many of these systems are run at a greater voltage (measured in kilovolts). Atmospheric operation eliminates the need for costly vacuum equipment and enables continuous product-type treatment [34]. A discussion of the most popular AP plasma systems is held.

#### 3.2.1. DBD

A high voltage is put between two metal electrodes to produce DBD plasma. The dielectric material, including glass, polymer, ceramic, or quartz, is used to cover one or both electrodes. The dielectric material's thickness varies and can be anywhere from 0.1 mm to several centimetres (Figure 1). The application for food treatment under package conditions, which involves CP creation inside the sealed package, opens up a wide range of options for the DBD system. This approach avoids postprocess contamination and extends the reactive species' window of opportunity for attacking microorganisms. The DBD reactor developed by Ziuzina et al. [35] was used for commercial production of food products. This prototype tested the *Escherichia coli* and *Listeria innocua* counts during the decontamination of cherry tomatoes under a continuous packaging system after using atmospheric cold plasma (ACP). The advantages of this technology include a spacious discharge surface, the ability to operate at ambient temperature, low maintenance expenses, and a potent sterilizing impact. Furthermore, it may be utilized in food in various stages.

#### 3.2.2. PJ

A variety of configurations are covered by the discharge known as PJ. Among the several design options, the two rings or coaxial (Figure 1) electrode configuration is the most common, in which the gas travels between the electrode pair. The ACP-PJ system based on a pilot scale was employed to inactivate the natural bacteria in particle foods (sesame, rice germ, and black pepper powder) and was recently developed [36]. Four PJs with an output voltage of 1 kV, a frequency of 30 Hz, and a length of the plasma emitted from the nozzle of 18 mm made up the PJ treatment compartment. The scientists found that 1.0, 1.4, and 1.4 log CFU/g of naturally occurring bacteria were inactivated in black pepper, rice germ, and sesame, respectively. The ACP-PJ system was utilized to alter the allergenicity of seafood. It was a single-phase, 220 V AC power source with a high frequency (50 kHz), 10 A electric current, and 7 kV DC output voltage [37]. To determine the impact of the CP on the stability of the total phenolic content and antioxidant properties, walnut samples were subjected to a test in a PJ reactor with a 1.5 cm space between the samples and the nozzle tip [38].

#### 3.2.3. CD

CD can be identified as a brilliant glow that is restricted in space close to pointed objects, edges, or thin wires when it is present in an electric field that is very irregular. An asymmetric electrode pair, such as a point and a plane, is produced whenever the electric field in a particular spatial position exceeds the threshold for the breakdown of the electric field. A weakly ionized plasma is produced when the gas breakdown strength is exceeded by the high electric field near the electrode (Figure 1). The multipoint-plate electrode arrangement is becoming increasingly popular for expanding the application of CAP in the food industry since it is capable of generating a more energetic and dense plasma compared to DBD. Employing a multipoint-plate reactor with a significant gap size of 70 mm [39] evaluated the effectiveness of CD on the main peanut allergens (Ara h 1 and Ara h 2).

## 4. PAW

The most popular plasma sources for producing PAW are PJ, gliding arc discharge, DBD, and surface microdischarge (SMD). The PAW is generated by exposing water to nonthermal plasma, a partially ionized gas consisting of ions, electrons, and neutral particles. The interaction between the plasma and the water leads to the formation of reactive oxygen and nitrogen species (RONS) in the water, which gives PAW its unique properties [40]. The plasma can be directly exposed to the surface of the water, or it can be generated in the gas phase and then brought into contact with the water. In some setups, water can be passed through a plasma zone. The duration of exposure to plasma affects the concentration of reactive species in the water. Longer exposure typically results in higher concentrations of RONS. Recently, several pilot units have been created for the manufacture of PAW. Andrasch et al. [33] produced PAW with a yield of 1 L/min using the plasma source PLexc2. Pemen et al. [41] constructed a pilot plant with a 0.5 L batch capacity for PAW manufacture. It is commonly known that using PAW, or water subjected to plasma discharge, is an alternate technique for microbiologically disinfecting food goods [42]. With its exceptional and broad antibacterial activity, PAW is an economical and environmentally friendly disinfectant that opens up new application opportunities in the processing of food, agriculture, and nutraceutical fields [42, 43]. Furthermore, other plasma-activated liquids (PALs) with good antibacterial properties included phosphate-buffered saline (PBS) [43]. The *Bacillus cereus* spores in 10^6^ CFU/mL were shown to be lowered by 1.63–2.95 log10 CFU/mL following PAW treatment at 55°C for 10–60 min [44]. Additionally, rice has been shown to benefit from the combined effect of PAW and low heat when combating bacterial spores. After 60 min of exposure to PAW at 40°C and 55°C, respectively, *B. cereus* spores in rice were deactivated by 1.55 and 2.13 log reduction [45]. For fruits and vegetables, PAW has an antifungal impact [46]. Zhou et al. [47] discovered that after 2 or 24 h at 15°C, PAW treatments only resulted in a 0.2 or 0.6 log CFU/mL decrease in the *Aspergillus flavus* spore population, although the samples' maximal metabolic activity reduced by 42.2% and 55.2%, respectively. Because of their unique structures, yeast and mold cells exhibit greater PAW resistance than bacteria [45]. Therefore, combining PAW with additional techniques like ultrasound and low heat could greatly enhance its antifungal efficacy [48]. Additionally, using PAW, the apparatus was used to investigate the effectiveness of pesticide degradation (chlorpyrifos and carbaryl) on grapes and strawberries. The authors noted that the carbaryl was reduced by 86% in grapes and 73% in strawberries, and chlorpyrifos was reduced by 79% in grapes and 69% in strawberries [49]. To improve the shelf life of red grapes, the study further combined ascorbic acid (AA) with a chitosan-based edible coating (CA) and treated the fruit with PAW (CA-PAW) before simulating transport vibrations [50]. In comparison to the control group treated with sterile deionized water, the CA-PAW treatment decreased microbial counts by 2.62 log10 CFU/g for bacteria, 1.72 log10 CFU/g for yeasts and molds, and 1.1 log10 CFU/g for coliforms, according to data from storage at 4°C for 20 days. According to Guo et al. [51], PAW effectively and time-dependently inactivated the bacteriophages T4, U174, and MS2. After 30 min of exposure, PAW and PALs (made with 0.9% NaCl solution and 0.3% H_2_O_2_ solution) could inactivate the Newcastle disease virus. Therefore, PAW is a viable alternative strategy to combat environmental and foodborne viruses because it is an environmentally benign technology.

## 5. CP as Pretreatment in Food Processing

Pretreatments are typically used to quicken processing by decreasing the temperatures and enhancing mass transfer with water [52, 53]. Recently, CP has been used as a pretreatment for drying corn kernels and chili peppers, resulting in improved drying rates [54]. Zhang et al. [55] used atmospheric air at a power supply of 750 W and pulse frequency of 20 kHz for a treatment time of 15–60 s, while Tabibian et al. [56] used atmospheric air with a power supply of 8 kV for a treatment time of 15–60 s, and Zhou et al. [57] used atmospheric air with a power supply of 750 W and pulse frequency of 20 kHz for a treatment time of 15–60 s to dehydrate chili, saffron pepper, and wolfberry, respectively. In the case of jujube slices, Bao et al. [3] performed pretreatment with CP for 15, 30, and 60 s, followed by hot air drying at 50°C, 60°C, and 70°C. Scanning electron microscope (SEM) analysis of the CP-treated sample showed that the slice surface topography changed due to the etching of larger cavities, which accelerated mass transfer by improving the drying rate and effective moisture diffusivity. Loureiro et al. [58] maintained different excitation frequencies of 200, 500, and 800 Hz using CP as a pretreatment for drying tucuma. SEM images revealed a change in the pretreated tucuma surface, which favored a faster drying rate and water diffusivity with reduced drying time.

The authors discussed how the application of plasma improved the extractability of active chemicals [59]. This theory is predicated on the fact that hydrophobic substances found on food surfaces lower surface tension, which in turn decreases the ability of hydrophilic substances to diffuse during extraction [60]. Moreover, the reactive gas species in plasma have enough energy to initiate chemical reactions that dissolve covalent bonds, liberating the phenolics that are attached to the food's structure [61]. Keshavarzi et al. [62] noted that the extraction of phenolic components from green tea leaves has been improved by CP of nitrogen gas. Because nitrogen has a stronger molecular binding force than other elements, it interacts with surfaces better, including cell walls, and releases more intracellular phenolic compounds [63]. CP has been utilized as preprocessing during grain heating to shorten processing times. It is known that CP interacts with proteins [64] and starches [65], creating active sites that can interact with other materials. According to Liu et al. [66], these new active sites encourage the grains to absorb water, which lowers the cooking temperature and duration. Several research studies assessed the impact of CP on the reduction of cooking time in different rice varieties, including brown rice [67], black gram [60], and Chinese milled rice [66]. Yong et al. [68] found that although the residual nitrite content was reduced, hams cured using plasma-treated water retained the same technical and sensory qualities as those treated conventionally prepared samples. Conventional methods of oil hydrogenation yield synthetic *trans*-fatty acids, which may diminish the nutritional value of goods [69]. Atomic hydrogen can be produced if plasma is created in an environment. Atomic hydrogen can interact with unsaturated fatty acid double bonds due to its high reactivity, which triggers the hydrogenation process [69]. In the conventional hydrogenation reaction, molecular hydrogen is separated and allowed to enter the unsaturated fatty acid through the use of a nickel catalyst in conjunction with high pressures and temperatures. Traditional hydrogenation can be replaced by cold plasma treatment, which produces hydrogenated oil free of trans fats at ambient temperature, AP, and catalyst-free methods [70].

## 6. Role of CP on Microbial Inactivation and Quality Improvement in Food Processing

**Figure 2:**
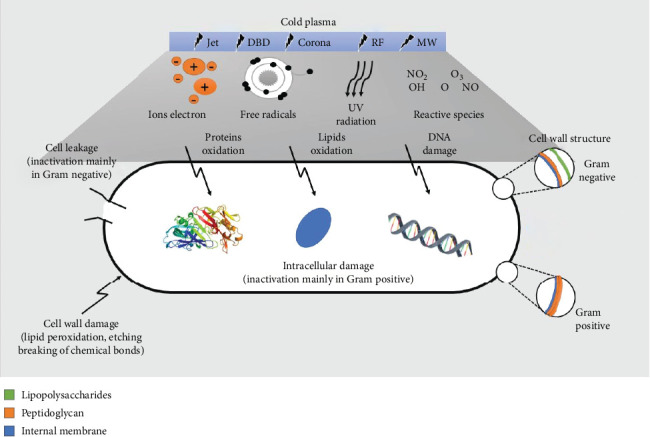
Inactivation mechanism for gram-positive and gram-negative bacteria using CP. CP, cold plasma.

CP technology has been extensively utilized in the electronics and textile industries. The food business remains in the early stages of plasma applications. CP has shown effectiveness in treating biofilms and decontaminating food items like poultry, meats, fruits, and vegetables [71]. CP has been employed for the surface treatment of various food items, including vegetables like fresh tomatoes [72], lettuce [73], and carrots [74]; fruits like apples [75], blueberries [76], and mangoes [77]; spices including red pepper [78], black pepper [79], and nuts [80]; legumes like chickpeas [81]; and animal products like ready-to-eat (RTE) meat, eggs, chicken, beef, lamb, and fish [82–85]. Numerous studies have demonstrated the efficacy of CP in the detoxification of liquid items like milk [86] and juices, including apple and orange juices [87]. The CP can effectively destroy pathogenic microbes like *E. coli* (O157:H7), *Salmonella*, *Shigella*, and *Listeria monocytogenes* that pose a threat to human health [88, 89]. *E. coli* is a major concern due to its contamination of food products. Lu et al. [90] reported that CP generated using atmospheric air with higher voltage (70 kV RMS (root mean square)) for shorter treatment durations (5 s) was efficient in inactivating *E. coli* and *L. monocytogenes.* The degree of microbial inactivation depends on factors like electrode material, air velocity, and relative humidity [91, 92]. Higher air velocity and lower relative humidity improved microbial inactivation efficacy. Han et al. [93] found that atmospheric air plasma with ROS was less effective in inhibiting *E. coli* than nitrogen plasma. The extent of microbial inactivation is dependent on membrane characteristics and treatment duration and needs to be optimized for maximum efficacy [94]. Recent findings of microbial inactivation using CP in various food products are shown in Table 1. The mechanism for inactivation in gram-positive and gram-negative bacteria is shown in Figure 2.

Glow discharge and DBD plasma are efficacious for the preservation of fruit and fruit juice [111, 112]. The demand for AP plasma in food processing has risen owing to its capacity for continuous product processing without requiring a vacuum [113]. The selection of the most suitable plasma application for processing products with optimal quality is contingent upon the chemical contents found in the fruits [114, 115]. Furthermore, vacuum plasma has had a beneficial impact on enhancing the vitamin C concentration in fruits, augmenting the nutritional quality of processed products, and altering the flavor and aroma of fruits [116]. Findings from numerous aforementioned and analogous investigations have demonstrated significant promise for CP technology in food applications, ensuring the preservation of safety and quality qualities.

### 6.1. Fruits and Vegetables With CP Treatment

The authors Gan et al. [117] reported that PJ treatment with chokeberry juice damaged the cell membrane of *E. coli*, leading to cytoplasmic leakage. However, for gram-positive bacteria, CP induces severe intracellular bacterial lysis with void of cell leakage [118]. Ziuzina et al. [119] employed DBD-CP to treat strawberries and cherry tomatoes for 10, 60, and 120 s, achieving microbial inactivation to nondetectable levels. Hosseini et al. [120] treated apple juice with DBD and ACP for 40 s with a 30–50 W power supply, resulting in a reduction of *E. coli* population to 3.98–4.34 log CFU/mL and compared the effectiveness of CP and thermal treatment in terms of microbial load and quality characteristics of sour cherry juice. They reported that CP treatment reduced *E. coli* populations by 6 log, while maintaining the phenolic content. In contrast, thermal treatment resulted in a 23% increase in phenolic content but decreased anthocyanin and vitamin C content by 26% and 77%, respectively. Timmons et al. [121] used surface DBD to inactivate *E. coli*, *S. enterica*, and *L. monocytogenes* in pecans and cherry tomatoes, with the inactivation efficiency decreasing with fewer treatment periods and increasing distances. Niveditha et al. [122] examined the response of *E. coli* to CP treatment in corn salad leaves and gel, concluding that the efficacy of CP treatment is dependent on the initial microbe count and the food commodity used. Dasan and Boyaci [123] evaluated the influence of CP on several food samples, including apple, tomato juice, orange, and sour cherry nectar. Santos Jr et al. [124] tested the efficacy of argon (Ar) gas in a CP reactor to inactivate *E. coli* in pumpkin puree, achieving a reduction of 3.62 log in 20 min, although this resulted in a reduction of carotenoids and pH content. Segura-Ponce et al. [125] discussed the impact of LP-CP on apple pieces, observing that the treatment had no important effect on microbial inactivation but did alter the surface wettability of the apple. A summary of the processing conditions for microbial inactivation on fruits and vegetables using CP is provided in Table 2. The experiment was performed in limes with inoculating *Penicillium digitatum* spore under manual conditions (10^6^ CFU/fruit), and then treatment was given with CAP at 30, 60, 90, and 120 s and compared with untreated samples [144]. The outcomes showed that extending the CAP exposure duration decreased the number of spores on the lime peel to less than 7 CFU/fruit in 120 s. Similarly, *Brassica chinensis* was stored for 10 days at 4°C ± 1°C to determine the impact of CP treatment on its antibacterial qualities and antioxidant activities [145]. The CP treatment was done on *B. chinensis for* 6, 9, and 12 min. Compared to the control group, *B. chinensis* treated for 9 min showed a 12.7% increased antioxidant capacity with microbial count reduction by 0.7 log CFU/g.

### 6.2. CP Treatment in Meat Products

**Figure 3:**
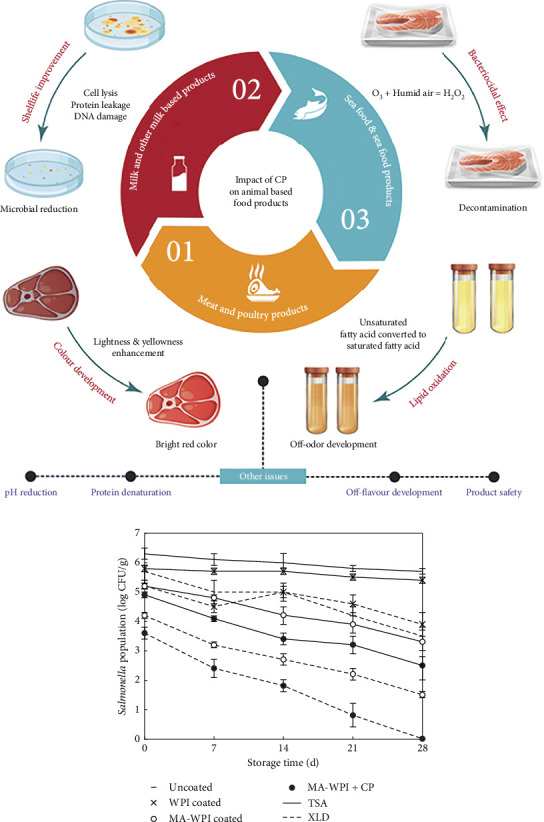
(a) Effect of CP on animal-based food products and (b) the growth of *Salmonella* in CBPM during storage at 4°C. (a) is adapted from Rajan et al. [146] and is an open-access article (copyright 2023 by authors) distributed under the terms and conditions of the Creative Commons Attribution-NonCommercial (CC-BY-NC) 3.0 unported licence, while (b) is adapted with permission (copyright 2023 Elsevier Ltd, Amsterdam, the Netherlands) from Jeon et al. [147].

Meat and its products are highly vulnerable to microbial growth and subsequent foodborne illness.  Figure 3a illustrates the impact of CP on food products derived from animals [146]. To address this issue, researchers have investigated the use of low-pressure helium and Ar plasma for bacterial inactivation of *E. coli* in meat [148]. The results showed that helium gas was more effective in reducing the bacterial population, achieving a 2.00 log cycle reduction in just 5 min of exposure, compared to Ar gas, which required 10 min for similar results. Voltage affects lipid oxidation, microbial reduction, and color properties of *Atlantic herring* (fish species), with a high concentration of polyunsaturated fatty acids, according to Albertos et al. [149]. Using 80 kV for 5 min during ACP-DBD therapy was efficient in suppressing *lactic acid* bacteria, *Pseudomonas*, total aerobic mesophilic, total aerobic psychotropic, and Enterobacteriaceae compared to a lower voltage (70 kV), which caused less redness loss and a slight increase in yellowness. CP treatment was also found to be effective in reducing microbial counts without affecting the properties of packaged beef [150]. Similarly, Rød et al. [151] demonstrated that the application of MAP (30%O_2_ + 70%Ar) gas to RTE beef during the in-package ACP treatment led to a maximum 1.6 log reduction in *L. innocua* cell counts. The *L. monocytogenes* was inactivated on RTE meat, including the surface of ham [152], beef jerky [153], fresh pork [154], and RTE meat bresaola [151]. ACP treatment can be applied to RTE beef products as a postdecontamination substitute [131]. In treating packaged chicken breast, an optimum condition of 39 kV for 3.5 min using atmospheric barrier discharge was found to be efficient in reducing the microbial population by 3.9 log CFU/cube of *E. coli* [150]. The author used the clove oil encapsulated with AP plasma, which was found to have a synergistic effect on the inactivation of *E. coli* and *Staphylococcus aureus* in beef jerky, resulting in a 7.5-log reduction in microbial count [155]. The synergistic effect was more pronounced for *E. coli* than *S. aureus*, likely due to the latter's thick layer of peptidoglycan. The studies demonstrate the potential of plasma to effectively reduce microbial counts in animal-based food products [146]. In this experiment, the antimicrobial efficacy of the coating of malic acid-incorporated whey protein isolate (MA-WPI) and the diffusion of MA in the skinless chicken breast processed meat (CBPM) was investigated about the in-package atmospheric DBD-CP treatment [147]. The *Salmonella* growth in four different CBPM conditions: uncoated, WPI-coated, MA-WPI–coated, and MA-WPI+CP treated at 4°C, which is depicted in Figure 3b. When evaluated by XLD, the growth of *Salmonella* in CBPM was inhibited at 4°C, regardless of the presence or absence of antibiotic treatment, indicating a typical growth pattern of mesophilic pathogens [156]. The *Salmonella* counts in MA-WPI–coated CBPM and MA-WPI+CP–treated CBPM dropped more quickly while being stored at 4°C compared to uncoated and WPI-coated CBPM. Specifically, during storage, the MA-WPI+CP–treated CBPM showed the fastest rate of decline in *Salmonella* counts (Figure 3b).

### 6.3. Plasma Effects Over Endogenous Enzymes

**Figure 4:**
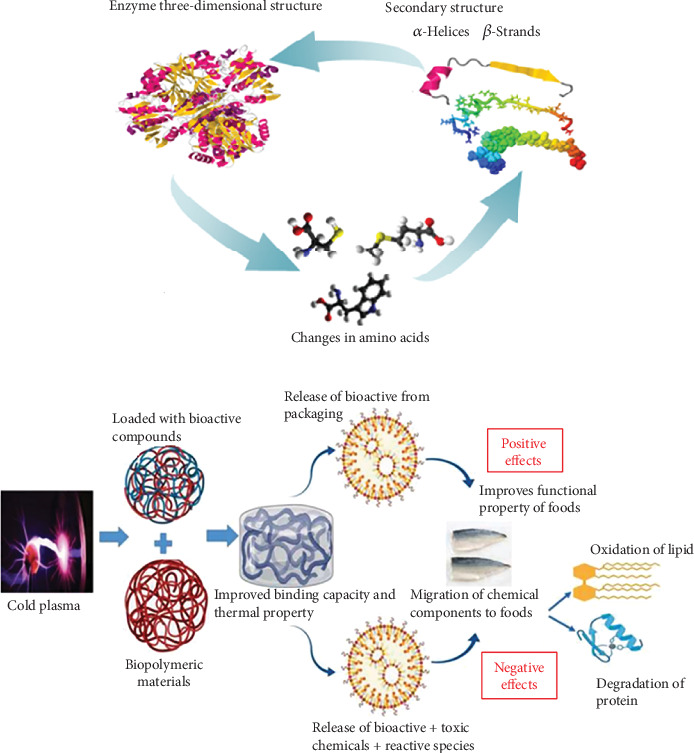
Effect of (a) CP in enzyme inactivation and (b) CP on food packaging material with positive and negative effects on food. (a) is adapted with permission (copyright 2018 Taylor & Francis, New York, United States) from Tolouie et al. [158], while (b) is adapted with permission (copyright 2020 Wiley Periodicals LLC., Hoboken, New Jersey, United States) from Ganesan et al. [159].

In addition to microbial infection, endogenous enzymatic browning is a significant component that contributes to food spoilage, resulting in unpleasant or unacceptable organoleptic qualities for ingestion. Specifically, fresh produce and related fresh-cut fruits and vegetables, such as litchi, peach, banana, apple, potato tissue, sugarcane, pear, strawberry, lettuce, Chinese yam, and mushroom, are the most frequently impacted items. The endogenous enzymes involved in enzymatic browning are primarily polyphenol oxidase (PPO), pectin methylesterase (PME), and peroxidase (POD), which oxidize phenolic compounds and generate off-flavors [157].  Figure 4a illustrates the mechanism of enzyme inactivation by plasma [158]. Enzyme inactivation occurs due to oxidation reactions that are accelerated by atomic oxygen and FRs [160]. Plasma treatment has been shown to reduce trypsin enzymatic activity, with plasma application altering the 3D structure of trypsin after cleavage of the peptide bond [161]. Similarly, Punia Bangar et al. [134] reported a reduction in PPO enzyme activity in guava pulp and fruit after treatment with plasma at 2 kV for 300 s. Pankaj et al. [162] studied the kinetic overinactivation of POD enzyme in tomatoes and found a decrease in enzymatic activity at different voltages after using atmospheric air DBD plasma. Attri et al. [163] observed that exposure to helium RF AP discharge improved lipase activity from 1 to 1.4 with increasing exposure time, possibly due to alterations in the molecular structure of the lipase, as confirmed by circular dichroism and fluorescence spectrum analysis. Finally, Abd El-Aziz et al. [164] investigated the changes in the antioxidant activity of enzymes in body tissues after treating meal moth larvae, finding significant improvements in the activity of catalase, lipid peroxide, and glutathione S-transferase, but no changes in glutathione POD activity. Furthermore, the sigmoidal logistic function was demonstrated to be the most suitable model for characterizing enzyme inactivation kinetics [165]. Ding et al. [143] utilized low-temperature AP plasma to examine the inactivation mechanism of lysozyme in aqueous solution, revealing that plasma-induced reduction of enzymatic activity was chiefly attributable to alterations in secondary structure and an increase in molecular weight. Bußler et al. [166] demonstrated that CP treatments effectively suppressed the activity of PPO and POD enzymes in fresh-cut apples and potatoes, allowing for an extension of posttreatment storage duration by 19 days. Surowsky et al. [167] previously provided insights into the inactivation mechanisms of PPO and POD within a model food system. The decline in the activity of PPO and POD enzymes resulted from plasma-mediated chemical alterations in their secondary structures, characterized by a decrease in alpha-helix content and an increase in beta-sheet regions. Furthermore, the activity of POD exhibited greater stability compared to PPO, as a plasma treatment duration of 180 s effectively diminished around 90% of PPO activity, while a 240-s treatment only inactivated 85% of PPO activity. In addition to PPO and POD enzymes, PME is a significant enzyme that has garnered considerable interest from researchers. PME, a prevalent cell wall-bound enzyme, significantly influences fruit ripening and impacts the shelf life of fresh food [168]. Nevertheless, data about the inactivation of PME through plasma therapy is exceedingly scarce. Tappi et al. [168] detected a minor albeit statistically insignificant decrease in PME activity in fresh-cut melon, despite a DBD plasma treatment duration of up to 60 min (30 min per side). It was thus hypothesized that various enzymes exhibit distinct resistances to plasma drugs, maybe attributable to their structural differences and the existence of isoenzymes.

Nonetheless, not all endogenous enzymes within food systems can be deactivated by cold plasma treatments. Xu et al. [169] showed that PAW can extend the shelf life of postharvest button mushrooms; however, the activity of superoxide dismutase (SOD) fluctuated during plasma treatment. Their findings demonstrated that in comparison to the control group, PAW treatments of varying durations (5, 10, and 15 min) followed by a 7-day storage period increased the levels of SOD. Zhou et al. [170] indicated that UV-C, aqueous ozone, and CP treatments enhanced the activity of SOD and CAT in blueberries; however, a gradual decline was noted after several days of post-treatment storage. Furthermore, CP exposure exhibited superior preservation effects compared to UV-C and aqueous ozone treatments. Comparable findings were documented by Puač et al. [171] in carrot calli. The plasma-mediated oxidative stress response and control of fresh produce may explain the increased activity of these endogenous enzymes. Consequently, additional research is required to elucidate the interaction and regulatory processes between the endogenous enzymes of fresh produce systems and CP, particularly at the molecular level.

### 6.4. Effects of CP in Low Moisture Food

Food products with a water activity (*a*_*w*_) < 0.85 are referred to as low-moisture foods (LMFs) [172]. However, the widespread attention they have received on food products is due to their frequent contamination with harmful germs [173].  Figure 4b shows the CP on food packaging material with positive and negative effects on food. Heat treatment works well to deactivate bacteria and other spoilage microorganisms in food, but it additionally causes loss of nutrients and pigments, which lowers the food's nutritional value and sensory properties [174]. Deng et al. [175] revealed that increased voltage and frequency resulted in increased inactivation of microorganisms by CP. Spices and food-grade rice starch had similar outcomes [176]. Charoux et al. [177] found that the vegetative cells of *B. subtilis* count in black pepper grains decreased noticeably by 1.16 ± 0.98 log (CFU/g) after 3 min of therapy at 15 kV and by 5.64 log CFU/mL after 5 min of exposure at 30 kV. Ahangari et al. [178] treated the walnut kernels with RF LP-CP at several powers and found that as power and time increased, the effectiveness of microbial inactivation was increased. The maximum reduction in the microbial population occurred when the treatment time was 20 min, and the power was 50 W. Total viable count, mold, and coliform were 0.89, 0.97, and 1.09 log CFU/g, respectively. Longer exposure time is not always preferable; however, an appropriate rise in CP treatment time will also boost the effectiveness of microbial inactivation. Hemmati et al. [179] investigated how the microbiological load of turmeric powder was affected by cold AP plasma. After 7 min of plasma exposure, a drop of 1.5 log CFU/g was seen in the amount of aerobic viable cells. Nevertheless, the plasma therapy's diminishing effect was only noticeable within the first 3 min of the procedure. The same findings have also been obtained by other investigations [36]. To maximize the efficacy of plasma therapy, it is crucial to understand the appropriate parameters [180]. Hertwig et al. [181] found that RF plasma therapy did not produce a comparable level of inactivation, and the microbial population of *S. enterica*, *Bacillus atrophaeus*, and *B. subtilis* spores was decreased by 4.1, 2.4, and 2.8 log after utilizing MW-assisted plasma therapy to treat pepper for 30 min. The distance from the source of plasma generation and the kind of ionized gas of the plasma system affected the deactivation rate of microbes. Sen et al. [182] treated *A. flavus* and *Aspergillus parasiticus* in hazelnuts using AP and low-pressure plasmas, respectively. Higher plasma power, frequency, and voltage were found to produce more inactivation in a shorter amount of time. Furthermore, the inactivation efficiency was significantly higher when air was utilized as a reactive gas than when nitrogen was employed, regardless of the type of plasma (AP or LP). Microbial growth is restricted in LMFs due to the lack of water required for microbial reproduction and growth. Nonetheless, a *a*_*w*_ < 0.6 is possible for some xerophilic spoilage fungi for their survival [183]. For example, *Aspergillus*, *Fusarium*, and *Penicillium* can contaminate LMFs such as wheat, rice, maize, and spices [184]. The Food and Agriculture Organization (FAO) of the United Nations reports that each year, fungal infections contaminate the world food supply by 25% [185], causing waste of food and adding economic loss to the nation. Mycotoxins are harmful metabolites that can be produced by some fungal diseases and are extremely harmful to human health [186]. CP mycotoxin destruction is considered to be a faster and more efficient approach than conventional treatments [187]. Diseases like carcinogenicity, teratogenicity, liver and kidney toxicity, and immunosuppression are among the common side effects of fungal infection [188]. Among the mycotoxins, the most common ones are *ochratoxin* (OTA), *nivalenol* (NIV), *aflatoxin* (AF), *fumonisin* (FB), *zearalenone* (ZEN), *deoxynivalenol* (DON), and so on [188]. There are many methods for restricting fungal growth and removing mycotoxin from processed food, but most of the methods are thermal methods, which ultimately hamper the product's quality. Basaran et al. and Amna et al. [189, 190] employed LP-CP to treat AF in hazelnuts and observed a 50% and 20% reduction of total AF (B1, B2, G1, and G2) after 20 min of therapy with air plasma and sulfur hexafluoride plasma, respectively. Makari et al. [191] used DBD-CP to compare the AF B1 degradation on pistachios and glass slides. It was found that CP therapy for 180, 120, and 60 s lowered the AF by 64.63%, 60.97%, and 35.41% on the glass slides and by 52.42%, 44.77%, and 32.31% on the pistachios, respectively. AFB1 degraded much more slowly on food than it did on glass slides due to the intricate nature of the food matrix. Durek et al. [192] found that after treating barley with CO_2_ plasma produced by plasma treatment, the amount of OTA produced rose to 72.9 ± 45.8 ng/g after 3 min of treatment from 49 ± 13.8 ng/g in the control group. Hoppanová et al. [193] found that after treating OTA with plasma for 60 and 90 s, OTA production rose dramatically in the first 4 days of incubation, but after 7 days, it was lower than the control. When comparing plasma-treated samples to untreated ones, the final OTA yield was lower in the plasma-treated sample. Process variables, including gas composition and storage duration, should be taken into account during actual processing. Janić Hajnal et al. [194] treated wheat flour for alternariol monomethyl ether and tentoxin toxins utilizing surface DBD-CAP at various treatment periods and plasma source distances. It was revealed that CP could lower the concentration of these three mycotoxins.

### 6.5. Effects of CP on Protein Allergenicity

Most allergens in the world are proteinaceous, frequently found in the proteins of plants and animals. Among the top food allergies are cow's milk, chicken eggs, tree nuts, peanuts, wheat, soy, fish, and shellfish [195]. The protein alterations are assisted in decreasing the allergenicity by the oxidation, aggregation, crosslinking, or fragmentation mediated by reactive species created during the high voltage cold atmospheric plasma (HV-CAP) therapy [196]. Soy protein isolate (SPI) was subjected to HV-CAP generated at different voltages (9, 10, and 11 kV) and durations (1, 2.5, 5, 7.5, and 10 min) in the study conducted by Meinlschmidt et al. [197]. The production of new protein bands at 50 kDa was seen together with the breakdown of the primary allergens of SPI, b-conglycinin (Gly m5), and glycinin (Gly m6), indicating the creation of insoluble aggregates following the plasma treatment. After receiving HV-CAP treatment for 10 min, there was a full (100%) decrease in SPI immunoreactivity. Additionally, the HV-CAP therapy reduced wheat allergenicity by 37% [198]. In dry, defatted peanut flour and the entire peanut, HV-CAP treatment at a voltage of 80 kV for varying treatment periods (0, 15, 30, 45, and 60 min) has been shown to lower “Ara h 1” in a time-dependent way [39]. The abovementioned samples' decreased allergenicity was linked to protein structural changes brought on by reactive species produced during HV-CAP treatment. Similarly, after HV-CAP treatment (40 kV, 12 kHz at 1, 2, 3, and 4 min), the soybean agglutinin (SBA) activity decreased in both the soymilk and SBA model systems. These alterations were ascribed to peptide bond cleavage, conformational modifications, oxidation of aromatic amino acids, and polypeptide breakdown, which resulted in SBA fragmentation and/or denaturation [199]. Meinlschmidt et al. [197] mentioned the application of direct and indirect treatment using CP on the SPI, which resulted in the disappearance of related protein bands from the SDS-PAGE profile, including *β*-conglycinin and glycinin. It was revealed that direct treatment controls allergenicity more quickly than indirect treatment. Regarding the direct treatment, the protein bands' strength started to drop after 2.5 min, and by 10 min, they practically vanished from the SDS-PAGE profile. Since SDS-PAGE can only detect soluble proteins, these results may point to a lower protein solubility brought on by plasma therapy. Protein aggregation via protein–protein cross-links was probably the cause of the decrease in protein solubility. In the same investigation, the newly generated protein band was seen in the SDS-PAGE profile after 5 and 7 min of direct treatment. It was proposed that the production of new soluble proteins was due to the aggregation of proteins by CP therapy as a result of the cross-linking of free amino acids with proteins [197]. In particular, it is thought that ROS and RNS cause the oxidation of side chains of amino acids and protein backbones, which results in protein breakdown via oxidation or cross-linking [200]. Ara h 1 is the major allergen present in peanuts. However, the thermal treatment elevates the allergenic potential of peanuts, resulting in the glycation of allergens. An experiment was performed by applying ACP treatment on peanut proteins before roasting with the addition of herein and glucose [201]. After 30 min of exposure to air or nitrogen plasma, Ara h 1's antigenicity dropped by 91% and 76%, respectively.

## 7. Effect of CP on Physical Properties of Food

Food color is a very important attribute that affects the perceptions of consumers toward the products. The color changes may be due to pigment degradation, including anthocyanin and chlorophyll [102]. In CP processing for food, the parameters, including the types of products like whole or piece products, solid, liquid, plasma processing conditions (power, time of exposure, input voltage, and gas used), and conditions of storage, are some crucial factors adding effect to the color. Additionally, processing with CP has also added a positive to the color of some food samples. Thirumdas et al. [202] have mentioned improvement in the whiteness and brightness index in brown rice with plasma treatment. Similarly, another research study investigated by Yong et al. [203] mentioned the use of CP to manufacture pork jerky without sodium nitrate additives. A similar color of redness in pork jerky was achieved by plasma processing while avoiding sodium nitrate. Therefore, recent studies have added significant results with the application of CP processing for product development by using natural sources by avoiding chemical preservatives [10, 204].

**Figure 5:**
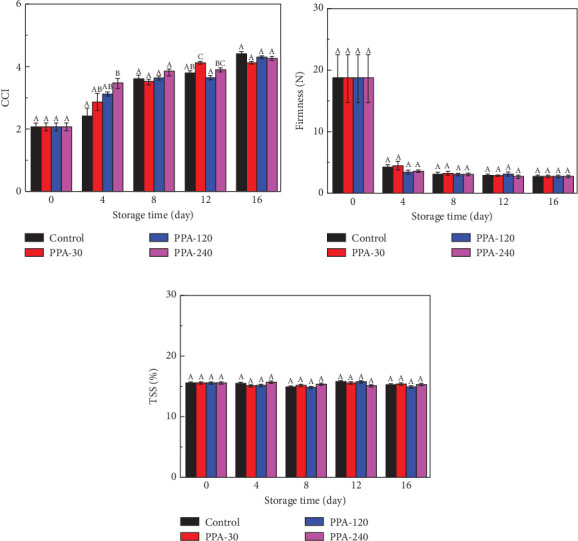
Effect of plasma-processed air (PPA) treatment on yellow peaches before and after storage of 4, 8, 12, and 16 days at 20°C. (a) Citrus color index (CCI), (b) firmness, and (c) TSS. The results were expressed as mean ± standard deviation of ≥ 3 independent replicates and error bars represent the standard deviation from mean values. Uppercase letters within the columns indicate insignificant differences (*p* > 0.05) among various treatments based on Tukey's honest significance test. This is adapted with permission (copyright 2022 Elsevier Ltd., Amsterdam, the Netherlands) from Wu et al. [205].

During storage, the yellow peach's citrus color index (CCI) value increased, which is related to variations in the number of carotenoid components throughout the fruit development period (Figure 5a) [205]. Indicating that plasma-processed air (PPA) treatment depended on the yellow color of the pericarp and this impact was favorable for the color of the yellow peaches, the CCI values of the PPA-240 treatment were substantially (*p* > 0.05) higher than those of the control at Day 4. At Day 16 of storage, there was no discernible difference between the CCI values of the control and any of the three PPA treatments (*p* > 0.05). The flavor of yellow peach fruit was distinctive, and texture was a key factor in determining how well-made yellow peach is and how it will fare in consumer markets. The fundamental reason for this is that at the climacteric peak of ethylene production, the firmness of peaches rapidly dropped (Figure 5b) [206]. Yellow peach softening slowed down from Day 4 to Day 16 and then stabilized. During storage, sugars and acids are used as substrates for respiratory metabolism, with corresponding changes in total soluble solids (TSS) [207]. Yellow peaches from all groups kept their TSS values consistent during 16 days of storage. After receiving PPA treatments, the TSS values of yellow peaches did not alter substantially (*p* > 0.05) (Figure 5c) from the control over the course of storage.

The texture retention of food products has been reported with CP treatment in food products. While considering fruits and vegetables, the effect of CP was not seen with differences in strawberries, melons, apples, and cherry tomatoes, whereas the firmness value was found to decrease after treatment for blueberries. The softness of fruits was due to mechanical damage at a higher air flow rate of plasma and elevated temperature. The CP treatment on strawberries packed in a modified atmosphere showed that the retention of firmness was higher in the O_2_ environment (65%O_2_ + 16%N_2_ + 19%CO_2_) than in the N_2_-enriched environment (90%N_2_ + 10%O_2_) [208]. These results revealed that plasma gas is a crucial gas to maintain the firmness of products. Similarly, the improvement of texture at higher O_2_ concentration and ozone treatment is reported by Pandiselvam et al. [209]. The results explained that firmness retention was higher with reducing the ripening rate and added stress at higher O_2_ concentrations. The treatment of legumes and grains with CP reduces the chewiness and hardness [210]. They also reported a reduction in cooking time for CP-treated products, which is beneficial in food industries. Similarly, the CP was treated with wheat flour and mentioned improvement in peak integral, dough strength, elastic modulus, and viscous modulus [211]. The flour protein's secondary structure was also affected by treatment with CP.

## 8. Effect of CP on Chemical Properties of Food

Researchers have conducted numerous studies on CP treatment by changing the pH value of the products [212]. The fluctuation of acidity and pH with plasma treatment is attributed to the interacting plasma gas with the moisture of the food sample. In the case of a solid food sample, the plasma species are reactive with surface water with the formation of the acid compound on the surface [213]. Feibel et al. [214] reported on nitric acid formation from RNS, including NO, due to acidification while performing treatment with air plasma. However, pH had shown no significant effects on CP treatment in food samples with buffering activity [215]. Consequently, the plasma effect on pH-stabilizing food ingredients is contingent upon various factors, including buffering capacity, living tissues, biological activity, and the potential for liquid generation from damaged tissues [216].

The CP effect on protein components in food products has been mentioned [217]. The protein denaturation with CP occurs due to the interacting effect of amino acid and plasma species with losing *α*-helix and *β*-sheet in the secondary structure of proteins [218]. The major factors, including processing parameters, the volume of sample, media of enzymes, reactive gas, types of plasma, and protein/enzyme, added the crucial role to proteins denaturation and inactivation of enzymes by CP. The CP was applied to the muscle protein of mackerel, and the study revealed a reduction in the immobilized water content located in the myofibrillar network [219]. Similarly, other investigations revealed a change in the protein structure in wheat flour due to the oxidizing effect of the sulfhydryl group by forming a disulfide bond, altering the functional and structural properties [220]. The ROS and RNS that are produced by CP have the ability to oxidize amino acid residues in proteins [221]. This results in the change of the structure of proteins through processes such as the creation of disulfide bonds, the oxidation of cysteine residues, and the fragmentation of protein chains. All of these modifications have the potential to bring about changes in the functionality of proteins, including variations in their solubility, digestibility, and texture. When lipid molecules are exposed to CP, they are capable of undergoing oxidation. Both unsaturated fatty acids and their double bonds are susceptible to damage by FRs, which can result in lipid peroxidation. Because of this, the sensory properties of food, such as its flavor and scent, can be altered, and in some instances, the nutritional value of the meal might be diminished because vital fatty acids are broken down or destroyed [222].

The CP-treated cashew apple juice was reported to degrade the reducing sugar, mainly glucose and fructose, as well as nonreducing sugar (sucrose) [223]. The long-term exposure to CP also increased the content of sucrose due to degraded oligosaccharides with its polymerization at a higher extent. Similarly, the fructose content was decreased with increasing sucrose content due to degraded oligosaccharides with polymerization at a higher extent in the CP-treated prebiotic drink of orange juice [224]. The higher uptake rate of water is reported in black gram [60], and the results were explained due to the rise of water binding sites with surface etching, which occurred due to protein and starch fragmentation by plasma species. Similarly, the same plasma was reported with decreasing cooking time for brown rice and was revealed with polar group incorporation within starch molecules [225]. They also mentioned improvement in gelatinization degree with plasma treatment. The other studies on the starch of rice [226] with lowering of amylose content, pasting and gelatinization temperature, degree of hydrolysis, and the tendency of retrogradation. In overall conditions, the CP processing depolymerized and cross-linked the starch with an added effect on functional, structural, and rheological characteristics [148].

Farooq et al. [219] revealed a minimum effect on vitamin C in radish, kiwifruit, and lettuce, respectively, whereas a reduction of 4% of vitamin C was seen after plasma treatment in fruits and vegetables. Similarly, the reduced content of vitamin C was observed in orange juice [227] and cashew apple juice [223]. The vitamin C reduction over plasma treatment occurs due to its reaction to ozone and other plasma species during the treatment process. The physical properties of the sample (whole or cut pieces), time of processing, and gases used were crucial factors in the degradation of vitamin C. However, the effect of CP processing should also emphasize other vitamins incorporated in food products by studying the mechanism of deactivations [228].

The oxidation of lipids poses a significant issue in muscle foods, resulting in adverse alterations such as reduced shelf life, unpleasant odor, altered taste, and discoloration. Lipid oxidation occurs through an FR chain reaction, potentially yielding fatty acyl peroxides or other oxidation byproducts [229]. Thiobarbituric acid reactive substance and peroxide value are methods applied to check lipid oxidation. The report revealed no major effect of CP on the oxidation of lipids in fresh and frozen pork, beef jerky, and raw pork [230]. However, Jayasena et al. [231] exhibited an increase in lipid oxidation in beef and pork that were subjected to a duration of 10 min. Similarly, lipid oxidation was improved in pork loin after treating it with O_2_-contained plasma gas. Albertos et al. [232] have also mentioned lipid oxidation by CP treatment in mackerel fillets. The CP treatment at 80 kV for 5 min was effective in improving the PV value from 6.89 to 37.57 meq. active oxygen/kg lipids and from 1.42 to 5.56 mmol of hydroperoxide/kg lipid. They also revealed a reduction in the content of oleic acid and eicosapentaenoic acid after CP treatments. The oxidation products of meat and dairy fats included the ozonides, carboxylic acids (9-oxononanoic acid, octanoic acid, and nonanoic acid), aldehydes (hexanal, pentenal, nonanal, and nonenal), and, along with hydroperoxides. The hydrogen plasma was used to manufacture the partially hydrogenated soybean oil to avoid the trans form of fatty acids. CP technology has shown a unique advantage in the hydrogenation process as the process is operational under ambient temperature and pressure while avoiding the requirement of a catalyst. Although the method is a potential alternative approach to catalytic hydrogenation, additional research needs to be performed to optimize the treatment process by evaluating the quality of hydrogenated oil obtained by CP treatment.

CP had no significant effects on apples, but it had a significant effect on cashew apple juice and blueberries [233]. These differences played a significant role in research to better evaluate the effect of CP on polyphenols present in food products at the molecular level. The antioxidant activity was not significantly affected by CP processing in the following products, including kiwifruits, red chicory, radish sprouts, and onion powder [234]. Similarly, in some research, a significant reduction of antioxidant activity in apples, cashew apple juice, and white grape juice after exposure to CP was observed [235]. Cavalcanti et al. [236] revealed a lowering of the antioxidant activity of the prebiotic drink of orange juice with the direct mode of application, whereas the results were insignificant while treated with the indirect mode. Therefore, the food product types, source of plasma generation, parameters of treatment, and exposure mode are the crucial factors of CP processing on the antioxidant properties of food products.

## 9. Effect of CP on Packaging Materials and Biofilm Formation

CP is a promising method for decontaminating packaging materials without causing internal damage to food products. Low-temperature gas plasma sterilization is a faster and safer process for packaging materials, including plastic bottles, lids, and films, without leaving any chemical residues or causing any adverse effects on the materials [237]. CP is particularly useful for sterilizing thermally sensitive packaging materials, such as ethylene and polycarbonate, as it operates at a lower temperature [236]. For edible packaging materials, a hydrophobic surface with lower energy at the surface is desired [238]. Ekezie et al. [31] investigated the O_2_ transfer properties of PET, LDPE, HDPE, and PP films coated with SiOx using CP and compared the results with computer models, finding lower diffusivity in the treated materials. Dong et al. [239] reported that plasma treatment of zein film led to a higher roughness, as observed by atomic force microscopy (AFM). The plasma-treated zein film had an RMS roughness of 100 nm, which was an improvement from the 20 nm in the untreated films, due to etching during the plasma treatment. Ganesan et al. [159] provide a schematic representation of the positive and negative effects of CP on food packaging material (Figure 4b). Han et al. [240] investigate the impact of storage on the inactivation of *L. monocytogenes* cells suspended in PBS solution following ACP treatment within the package. They used air as the plasma gas for 1 min during the in-package ACP treatment. Following 1 and 24 h of post-treatment storage, they found a 4.26 and 6.02 log CFU/mL reduction in *L. monocytogenes*, respectively. The earlier investigation assessed how in-package ACP treatment affected the decrease in *L. monocytogenes* at the surface of the ham after storage for 24 h and 7 days. The findings revealed that *L. monocytogenes* cells decreased by an additional three logarithmic units after storage of 7 days, as opposed to 24 h for samples exposed to ACP for 10 min [241]. Similarly, the hurdle approach was applied for the decontamination of fresh/cut vegetables wrapped in plastic vessels using CP(HCP) and H_2_O_2_ [140]. *E. coli* O157:H7, *L. monocytogenes*, and native aerobic bacteria were all reduced in mixed vegetables by 1.5, 1.3, and 1.4 log CFU/g after 3 min of HCP treatment.

In current times, biopolymer-based packaging material is used for the packaging of food materials. Several research studies have been carried out on the effect of CP on biopolymer-based packaging materials. Cools et al. [242] used the biodegradable films of polybutylene terephthalate (PBT) and polyethylene oxide terephthalate (PEOT) treated with DBD plasma including N_2_, He, Ar, and air. According to reports, the PEOT/PBR film's surface was maintained with a more uniform surface after the He and Ar plasma treatment removed the sharp peaks. Honarvar et al. [243] found that using atmospheric CP enhanced the way carboxymethyl cellulose adhered to polypropylene films. Similarly, Fazeli et al. [244] observed a notable improvement in the adhesion of thermoplastic starch and cellulose fiber treated with air plasma. Other research has demonstrated that plasma treatment improved the adhesion between various polymer layers, such as in films made of polyethylene terephthalate and polypropylene that were constructed using chitosan, which was loaded with various preservatives [245], and low-density polyethylene coated with collagen-containing cinnamaldehyde [2, 246]. Contact angle reflects a material's wettability and hydrophilicity by showing a liquid drop's ability to spread out and stick to its surface [247]. The surface's propensity to reject water increases with higher contact angle values. This increases the surface tension, polarity, and surface-free energy of the film surface, ultimately making it more wettable and hydrophilic [248]. When SF6 plasma was applied to corn starch thermoplastic sheets, the contact angle values climbed [249]. The voltage, exposure duration, and plasma gas composition affect the contact angle. The contact angle is a crucial determinant of a film's suitability for packaging applications [250]. A film's wettability has an impact on the surface of the film in terms of adhesion, coating, printing, and friction properties [251]. Goiana et al. [247] coated the corn starch-based film with DBD plasma and found that tensile strength (TS) increased considerably when treatment time was increased from 0 to 20 min. Similarly, Sheikhi et al. [249] also showed that when the treatment period was prolonged from 0 to 12 min, the TS of Ar plasma-treated starch film increased considerably. The use of carbonyl groups as cross-linking groups, combined with additional plasma exposure, encouraged hydrogen bonding, which enhanced the film's structural integrity due to the rise in TS [247]. Similarly, Chen et al. [252] modified zein and chitosan-based composite films by applying CP for 60 s and found that the TS and EAB considerably improved in comparison to zein or the zein/chitosan composite without the plasma treatment. TS and EAB dropped as a result of the zein/chitosan composite's lower compactness due to the high energy in the plasma and the lengthier 90-s film treatment. Pankaj et al. [253] examined the effects of ACP treatment at high voltage (80 kV) on the thermal characteristics of starch films made of rice, potato, corn, and tapioca. It was stated that the treatment gave the films a highly ordered structure, which resulted in a higher glass transition temperature (*T*_*g*_) and Δ*H*_*m*_ than in the control films. Furthermore, it was noted that the *T*_*g*_ and *ΔH_m_* values were lowest for the rice starch with the lowest amylose level and greatest for corn starch with a high amylose content [253]. Dong et al. [239] reported that the denaturation temperature (*T*_den_) was raised by an atmospheric CP treatment lasting 60 s, according to DSC data that examined the thermal characteristics of zein films. This effect may have been caused by the cross-linking of polar molecules produced at the surface of the film. The water vapor permeability (WVP) of the packaging material is a crucial factor in figuring out its moisture barrier quality, which affects packed food's shelf life and quality. The WVP of starch/PCL and starch/PLA composite films was reduced by roughly 94% after CP treatment [254]. Owing to the improved adhesion characteristics brought about by plasma treatment, various composite films, such as zein/chitosan [252], zein/PLA [11], and whey protein concentrate/wheat cross-linked starch [255] films, demonstrated reduced WVP. Dong et al. [239] treated zein film with atmospheric CP and found that, in comparison to control films, WVP dropped by about 24%. Oxygen regulates the development of several reactions, such as oxidation, which involves components that give food products their color and scent [256]. Ledari et al. [256] mentioned that oxygen permeability (OP) dramatically dropped when air, O_2_, N_2_, air, Ar, and ethanol–Ar mixture were applied to the gelatin film. Similarly, when exposed to air and Ar plasma, the OP significantly decreased in the whey protein film and gluten film [257]. Sheikhi et al. [258] treated the starch film with air and O_2_ plasma at low pressure and saw a considerable drop in OP.

The way that the PRS interacts with the biofilms is comparable to how free-living cells interact with one another [259]. Stopforth et al. [260] found that compared to free-living planktonic bacteria, *L. monocytogenes* biofilms in beef exhibited a higher resilience to antimicrobial treatments. The nutrient-rich environment within the EPS matrix allows the biofilm cells to multiply and become resistant to direct attacks by plasma species [261]. The inactivation procedure is challenging because free-living and planktonic bacteria differ in their phenotypic alterations and functional characteristics [262]. The age of the biofilm influences the efficacy of CP as a microbicide against bacterial biofilms [263] and is affected by environmental growth conditions [1]. For the removal of biofilms, several CP-mediated antivirulence routes exist [264]. The CP works with ROS-mediated cell membrane damage [265]. Through the outermost cell membrane, the plasma-mediated ROS and RNS can permeate and cause oxidative stress, lesions, and lysis of the cell wall due to electrostatic phenomena. Cell leakage is the result of the electrostatic forces generated by the charged particles rupturing the cell membrane [266]. The next step is the entrance of ROS into the cell, which causes more chemical destruction of the macromolecules [267]. Single and double cleavage by ROS breaks down DNA into smaller oligonucleotides, which might lead to DNA mutations [1]. Neutral H and OH radicals are produced when the water layer surrounding the biofilm cells interacts with CP. Intracellular oxidative stress is caused by secondary FRs that are produced by further reactivity with the cell membrane [268]. The study explored the biofilm-inhibitory effect and antibiofilm mechanism of DBD-CP on *Pichia manshurica*, which is associated with biofilm spoilage in fermented food. With plasma treatment (80 kV, 50 Hz) for 4.5 and 7.5 min, viable counts were reduced by 2.38 and 5.11 log CFU/mL, respectively, and the rate of biofilm formation was reduced to 73% and 48% of the control group, respectively [269]. Similarly, the experiment was designed to evaluate the effect of PAW on the biofilm formation characteristics of food isolate *Salmonella enteritidis* [270]. Studies of the three main components of biofilm extracellular DNA (eDNA), protein, and carbohydrates confirmed these findings. These elements were substantially less prevalent in the 24 h biofilms made by *S. enteritidis* cells treated with PAW than in the biofilms made by untreated cells.

## 10. Effect of CP Pretreatment on Energy Consumption

Recent studies indicate that CP pretreatment can substantially enhance drying efficiency by optimizing energy utilization. Shishir et al. [4] observed that the treatment of shiitake mushrooms with CP and CP-activated water (CPAW) required 0.43 and 0.22 kWh/kg of energy, respectively. They reported that the hot air drying of untreated, CP, and CPAW samples required 53.40–62.00, 42.6–51.0, and 46.5–53.75 kWh/kg, respectively, contingent upon the drying temperature (50°C, 60°C, and 70°C). According to the data, CP pretreatment resulted in a minimal energy input while significantly reducing overall energy consumption (from 53.4 to 42.6 kWh/kg sample) by decreasing drying time (from 13.1 to 10.6 h). The plasma treatment in this study ranged from 62.00 to 43.03 kWh/kg, typically averaging 0.43 kWh/kg, yielding approximately 40% energy savings relative to untreated conditions, as documented in the paper. A separate study indicated that CP pretreatment of saffron reduced the 369 kJ necessary for CP treatment by 60 s, resulting in a 40% decrease in overall energy consumption relative to the untreated sample. The total energy required for drying was reduced from 45.72 to 33.70 kWh after 50 s of treatment. The power consumption for CP pretreatment varied from 0.0008 to 0.005 kWh. The drying duration was reduced from 9600 to 3600 s by the application of CP and hot-air drying, as demonstrated by Ranjbar Nedamani and Hashemi [271]. This resulted in an 85-time reduction in energy consumption when compared to traditional drying. In contrast to other treatment procedures, plasma's energy cost is less expensive [272]. Nevertheless, plasma treatment will result in substantial savings on dehydrating energy expenses. It is crucial to bear in mind that the energy efficacy of the drying process may be influenced by the layout of the plasma devices.

## 11. Effect on Environment and Economic Feasibility of CP

The objective of food processing industries is to satisfy consumer demand for high-quality foods while enhancing their economic standards and achieving net profits. In recent years, food processing businesses have concentrated on energy consumption and energy conservation. This can only be accomplished through the utilization of unconventional developing technologies, as thermal preservation approaches necessitate substantial water resources and incur additional expenses for wastewater management. Numerous publications indicated that the energy efficiency of unconventional technologies surpassed that of thermal processing. In numerous developing nations, food processing industries are consistently assessing the implementation of nonconventional technologies, as these technologies are both energy-efficient and water-conserving, particularly in light of the water scarcity that has already arisen in many parts of the world, especially in developing countries such as India. Schlüter et al. [273] indicated that plasma treatment is considered a viable alternative to other chemical or physical treatments, such as HPP, pulse electric field (PEF), and irradiation. The advantages of plasma processes include high efficiency at low temperatures, accurate plasma generation tailored for specific applications, just-in-time creation of active agents, minimal impact on the internal product matrix, solvent-free and water-free application, absence of residues, and resource efficiency. The primary benefit of CP technology for the removal of volatile organic compounds (VOCs) from the food industry, which are toxic and detrimental, lies in its comparatively low energy consumption and moderate cost relative to traditional air treatment methods. Furthermore, it effectively treats air with low concentrations of VOCs at relatively low operating temperatures [10].

## 12. Limitations and Challenges of CP

The use of CP as a method for decontaminating food is hampered by the inherent limits of chemicals. On a laboratory scale, the application of CP in food is still being done. Insufficient study on food quality characteristics and the uncertain nature of the operating conditions of the CP technology are the key factors that are preventing its adoption [274]. Reactive plasma species, such as FRs, can provoke the autooxidation of lipids and lipid constituents in the diet, leading to various irreversible alterations [275]. Studies have demonstrated the influence on physiological activity and sensory attributes. Researchers have demonstrated that CP treatment influences color variations in tomatoes and carrots, as well as photosynthetic activity in cucumber and fresh corn salad leaves [276]. Lee et al. [140] noted decreases in vitamin C in CP-treated cucumbers, while Zargarchi et al. [277] observed diminished antioxidant activity on pear surfaces, and Subrahmanyam et al. [278] recorded reductions in the pulp and peel of apples. The restricted penetration of CP is a significant obstacle to its use, as microorganisms residing beyond the effective penetration depth may remain unaffected. Additional limits of CP may encompass a reduction in the hardness of fruits and vegetables, discoloration, and increased acidity. The efficacy of CP treatment is significantly affected by the surface topography of the sample [274]. A significant constraint in the commercialization of this technology is the substantial capital expenditure needed. Moreover, increasing capacity necessitates a substantial discharge area, resulting in considerable additional expenses. These financial obstacles can impede the extensive use of CP technology across many sectors. Addressing obstacles, such as identifying cost-effective scaling solutions, will be essential for its wider use. The absence of explicit legislative mandates for CP technology may contribute to its restricted implementation in industrial applications. Regulatory standards and guidelines are crucial for guaranteeing the safety, quality, and compliance of emerging technology, particularly in their respective industries. The lack of clear regulations might generate uncertainty and hesitation among prospective users and investors. Establishing explicit legal frameworks and standards for CP technology could accelerate its transition into industrial applications and encourage its responsible and safe utilization. Future studies should concentrate on a comprehensive analysis of CP interactions with food components and their impact on food quality. A comprehensive cost study for large-scale uses of CP is essential. This is mostly dictated by the quantity of electricity and the selection of feed gas.

Successful employment in the food business presents numerous obstacles in the application of CP. The future of CP in the food sector hinges on elements such as comprehension of reactive plasma species, inactivation processes, and other detrimental impacts. Understanding more about how it works will be very important for making the best use of it in food processing. This is because UV light and the chemical actions of radicals and reactive species are the main things that make it antibacterial. The effects of CP treatments on taste, flavor, fragrance, and other sensory characteristics must be determined. Additionally, it is necessary to determine whether it leaves a chemical residue, poses potential toxicological effects on food, or induces allergenicity. Compiling literature on the effects of CP on various food product categories would facilitate regulatory approval by authorities and increase public knowledge for broader acceptability. Currently, CP serves as a chemical-free method for small-scale processing, making it a prime choice for further investigation in the processing of organic foods.

## 13. Conclusions and Future Prospectives

CP technology has seen extensive use in a variety of food applications over the course of the past decade. There are a multitude of benefits, including the reduction of microbiological contamination; the enhancement of qualities such as hydration, rheology, and emulsification; and the preservation of the nutritious content while simultaneously reducing the antinutritional content. This study underscores the ongoing research on CP technology in the context of food processing. We can conclude that the application of CP effectively enhances the quality of consumable products by extending their shelf life; we have observed favorable outcomes in terms of both quality and microbial activity across various food categories; additionally, consumer awareness and a preference for healthier foods are driving an increasing demand for raw or nonheat-treated foods. CP is highly efficacious in diminishing food spoilage bacteria, viruses, and fungi; hence, it augments food safety. In addition to prolonging the shelf life of food products, CP treatment effectively preserves the physicochemical features of treated items, including color, texture, flavor, and chemical constituents. CP functions as a pretreatment for drying food products, ensuring food safety and quality, inhibiting biofilm formation, reducing protein allergenicity, and efficiently sterilizing food packaging materials. Similarly, CP is proficient at decontaminating LMF items. Furthermore, CP proficiently alters biopolymer-based food packaging materials. This research enhances the comprehensive understanding and prospective applications of CP technology in food processing. It is essential to delineate the procedures for various food items, and comprehending the mechanism of action is a crucial step in optimizing technology for specific applications within the food processing sector.

## Figures and Tables

**Table 1:** Studies dealing with inactivation of microorganisms by CP.

**Associated microbes**	**Substrate**	**Plasma types**	**Exposure time**	**Log reduction**	**References**
*Escherichia coli*	Almonds	Dielectric discharge	30 s	1.8–5	Lee et al. [95]
*Aeromonas hydrophila*	Lettuce	Cold oxygen plasma	5 min	5	Hu et al. [96]
*Aspergillus flavus*	Pepper powder	Microwave-cold plasma technology	20 min	2.5	Esmaeili et al. [97]
*Salmonella typhimurium*	Tomatoes	DBD	300 s	3.8	Kongboonkird et al. [98]
Yeast/mold	Strawberries	DBD	5 min	Upto 3	Shabani et al. [99]
*Staphylococcus aureus*	Orange juice	DBD	25 s	> 5	Liao et al. [100]
*E. coli*	Bacon	Atmospheric-pressure plasma	90 s	2–3	Sudarsan and Keener [101]
*S. typhimurium*	Strawberry	Cold oxygen plasma	15 min	1.76	Laroque et al. [102]
*Saccharomyces cerevisiae*	Mango and melon skin	Atmospheric plasma jet	25 s	1–3	Asghar et al. [103]
*B. cereus* (vegetative cells)	Rice matrix	Low-pressure cold plasma	12.5 min	1.9–3.31 UFC/mL	Valdez-Narváez et al. [104]
*B. cereus* (vegetative cells)	White rice	Atmospheric plasma DBD	20 min	5 log cycle	Lee et al. [105]
*B. cereus* (vegetative cells)	Brown rice	Atmospheric plasma DBD	20 min	1.9 log cycle	Lee et al. [105]
*B. cereus* and *E. coli*	Red pepper powder	Atmospheric plasma DBD	15 min	1 log CFU/mL	Kim et al. [106]
*B. cereus* spores	Dry pepper grains	Atmospheric pressure plasma jet	20 min	1.37 log CFU/g	Liu et al. [107]
*Listeria monocytogenes*	Fresh shrimp	ACP	30 min	0.94 log CFU/g	Liu et al. [108]
*L. monocytogenes*	Frozen shrimp	ACP	30 min	1.68 log CFU/g	Liu et al. [108]
*Vibrio parahaemolyticus*	Fresh shrimp	ACP	30 min	1.26 log CFU/g	Liu et al. [108]
*V. parahaemolyticus*	Frozen shrimp	ACP	30 min	2.0 log CFU/g	Liu et al. [108]
*A. flavus* and *A. parasiticus*	Hazelnuts	Atmospheric pressure fluidized bed plasma	5 min	4.50 log (CFU/g) in *A. flavus* and 4.19 log (CFU/g) in *A. parasiticus*	Dasan et al. [109]
*A. flavus*	Corn kernels	Low-temperature plasma treatment	90 s	4.03 log (CFU/g)	Li et al. [110]

Abbreviations: ACP, atmospheric cold plasma; CP, cold plasma; DBD, dielectric barrier discharge.

**Table 2:** Inactivation of microorganisms on fruits and vegetables by CP.

**Food types**	**Types of plasma**	**Microorganisms**	**Process conditions**	**Changes**	**References**
Granny Smith apples	Plasma jet	*Listeria monocytogenes*	40 Hz, 36 kV, and 5 min	Apple surface structure changes	Ukuku et al. [126]
Unpeeled almond	Diffuse coplanar surface barrier discharge	*Salmonella*	20 kV, 15 kHz, and 15 min	Color change	Hertwig et al. [127]
Groundnuts	Radio frequency plasma	*Aspergillus parasiticus* and *A. flavus*	60 W and 13.56 MHz	No change	Devi et al. [128]
Strawberries	DBD	Yeasts and molds and aerobic bacteria	50 Hz, 60 kV, and 5 min	No change	Figueroa-Pinochet et al. [129]
Mangoes and melons	CAP	*E. coli* and *Saccharomyces cerevisiae*	12–16 kV, 30 kHz, and 1 min	No change	Sun and Wang [130]
Mandarins	Microwave-powered CP	*Penicillium italicum*	2.45 Hz, 0.7 kPa, 900 W, and 10 min	Peel has higher antioxidants and phenolics	Pant et al. [131]
Apple	DBD	*E. coli O157:H7*	150 W, 12.7 kHz, air, and 120 min	Reduction of antioxidant	Punia Bangar et al. [132]
Golden apple	Gliding arc CP	*Salmonella Stanley* and *E. coli O157:H7*	15 kV, 60 Hz, and 3 min	No change	Deshmukh et al. [133]
Cabbage	Microwave-powered CP	*L. monocytogenes*	400–900 W, 667 kPa, and 1–10 min	No change	Punia Bangar et al. [134]
Onion powder	Microwave plasma	*A. brasiliensis* and *E. coli O157:H7*	400–900 W, 2.45 GHz, 0.7 kPa, 1 L/min, and 10–40 min	No change	Kim et al. [135]
Tomato	DBD	*E. coli ATCC 25,922*	15 Hz, 60 kV, and 5–30 min	No change	Punia Bangar et al. [132]
Radish	Microwave-powered plasma	*S. typhimurium*	900 W, 667 Pa, and 10 min	Reduction in the moisture content	Bora et al. [136]
Spinach	DBD	*E. coli ATCC 25,922*	50 Hz, 12 kV, and 5 min	No change	Sudarsan and Keener [101]
Corn	High voltage atmospheric CP	Aflatoxin*s*	90 kV, 50 Hz, and 10 min	No change	Nguyen et al. [137]
Pumpkin	Intermittent corona discharge plasma jet	*E. coli ATCC 25,922*	17 kV and 20 min	Decrease in pH	Santos Jr et al. [124]
Blueberries	Indirect atmospheric pressure plasma jet	Reduce spoilage organisms including yeast (0.9 log CFU/g) and mold (3.27 log CFU/g)	549 W and 47 kH Distance: 7.5 cm Time: 0, 15, 30, 45, 60, 90, and 120 s Store for 1, 2, and 7 days period	Remarkable reduction in APC from 0.8 to 1.6 log CFU/g and 1.5 to 2.0 log CFU/g after 1 and 7 days of storage, respectively, for all treated samples at all times	Lacombe et al. [138]
Carrot	Atmospheric air	Targeted microorganisms included *E. coli*, *P. marginalis*, *P. carotovorum*, *L. innocua*, *S. aureus*, *B. atrophaeus Nakamura*, and *C. albicans*	Sample placed in a glass bottle at a fixed distance: 25 cm; time: 7 s; gas temperature: 4000 K; post-treatment: bottle closed; 5, 10, and 15 min	*E. coli* and *P. carotovum* showed linear inactivation till the detection limit after 15 min	Schnabel et al. [139]
Mixed vegetables (kale, cabbage, and paprika)	CP + H_2_O_2_		3-min treatment	Reduced the indigenous aerobic bacteria, *E. coli* O157:H7, and *L. monocytogenes* in mixed vegetables by 1.6, 1.2, and 1.3 log CFU/g, respectively	Lee et al. [140]
Cabbage	In package CP + H_2_O_2_	*Salmonella*	3 min	Reduced by 1.8 log CFU/g at 10% H_2_O_2_	Kim and Min [141]
Romaine lettuce	Atmospheric dielectric barrier discharge CP	*E.coli*, *Salmonella*, and *L. monocytogenes*	35 kV for 5 min	Reduced the population of *E. coli* O157:H7, *Salmonella*, and *L. monocytogenes* by 1.1, 0.4, and 1.0 log CFU/g, respectively	Min et al. [142]
Apple juice	CP	*Alicyclobacillus acidoterrestris* spores	18 min	4.4 log reduction (CFU/mL)	Ding et al. [143]

Abbreviations: CAP, cold atmospheric plasma; CP, cold plasma; DBD, dielectric barrier discharge.

## Data Availability

The data is contained within the article.

## Nomenclature

AP: atmospheric pressure
CAP: cold atmospheric plasma
CP: cold plasma
DBD: dielectric barrier discharge
PJ: plasma jet
MW: microwave
CD: corona discharge
SMD: surface microdischarge
RONS: reactive oxygen and nitrogen species
FRs: free radicals
PAW: plasma-activated water
RNS: reactive nitrogen species
ROS: reactive oxygen species
RF: radio frequency
FAO: Food and Agriculture Organization
SPI: soy protein isolate
AFM: atomic force microscopy
HV-CAP: high-voltage cold atmospheric plasma
CCI: citrus color index
PPA: plasma processed air
SBA: soybean agglutinin
TSS: total soluble solids
TS: tensile strength
WVP: water vapor permeability
OP: oxygen permeability
PALs: plasma-activated liquids
PBS: phosphate-buffered saline
AA: ascorbic acid
CBPM: chicken breast processed meat
LMFs: low moisture foods
CCP: capacitively coupled plasma
ICP: inductively coupled plasma
PEOT: polyethylene oxide terephthalate
PBT: polybutylene terephthalate
